# The Diagnostic Significance of CXCL13 in M2 Tumor Immune Microenvironment of Human Astrocytoma

**DOI:** 10.3389/pore.2022.1610230

**Published:** 2022-04-28

**Authors:** Shu-Jyuan Chang, Chia-Te Chao, Aij-Lie Kwan, Chee-Yin Chai

**Affiliations:** ^1^ Department of Pathology, College of Medicine, Kaohsiung Medical University, Kaohsiung, Taiwan; ^2^ Department of Pathology, Kaohsiung Medical University Hospital, Kaohsiung, Taiwan; ^3^ School of Medicine, College of Medicine, Kaohsiung Medical University, Kaohsiung, Taiwan; ^4^ Department of Neurosurgery, Kaohsiung Medical University Hospital, Kaohsiung, Taiwan; ^5^ Department of Surgery, Faculty of Medicine, College of Medicine, Kaohsiung Medical University, Kaohsiung, Taiwan; ^6^ Institute of Biomedical Sciences, National Sun Yat-Sen University, Kaohsiung, Taiwan

**Keywords:** immunohistochemistry, gliomas, M2 macrophages, astrocytoma, CXCL13

## Abstract

**Background:** CXCL13 may act as a mediator of tumor-associated macrophage immunity during malignant progression.

**Objective:** The present study clarifies the clinicopathological significances of CXCL13 and its corresponding trend with M2 macrophage in human astrocytoma.

**Methods:** The predictive potential of CXCL13 was performed using 695 glioma samples derived from TCGA lower-grade glioma and glioblastoma (GBMLGG) dataset. CXCL13 and M2 biomarker CD163 were observed by immunohistochemistry in 112 astrocytoma tissues.

**Results:** An in-depth analysis showed that *CXCL13* expression was related to the poor prognosis of glioma patients (*p* = 0.0002) derive from TCGA analysis. High level of CXCL13 was detected in 43 (38.39%) astrocytoma and CXCL13/CD163 coexpression was expressed in 33 (29.46%) cases. The immunoreactivities of CXCL13 and CXCL13/CD163 were found in the malignant lesions, which were both significantly associated with grade, patient survival, and IDH1 mutation. Single CXCL13 and CXCL13/CD163 coexpression predicted poor overall survival in astrocytoma (*p* = 0.0039 and *p* = 0.0002, respectively). Multivariate Cox regression analyses manifested CXCL13/CD163 phenotype was a significant independent prognostic indicator of patient outcome in astrocytoma (CXCL13, *p* = 0.0642; CXCL13/CD163, *p* = 0.0368).

**Conclusion:** CXCL13 overexpression is strongly linked to CD163+ M2 infiltration in malignant astrocytoma. CXCL13/CD163 coexpression would imply M2c-related aggressive characteristics existing in astrocytoma progression could also provide predictive trends of patient outcomes.

## Introduction

Astrocytoma is the most common malignant primary glioma in brain tumors. In terms of clinical pathological evaluation, astrocytoma can be generally classified into grades one to four. Grade I tends to be benign and grade II is a low-grade tumor, while grades III and IV are defined as high-grade tumors, which indicate poor patient prognosis (1). The course of treatment and treatment efficiency depends on the location, size and malignancy of stellate cell tumors. Unfortunately, irreversible damage usually occurs, leading to miserable aphasia or limb dyskinesia. A closed system comparison with other tumors is possible in the case of astrocytoma due to the presence of the blood-brain barrier, and as a result, local micro-environmental stress or benefits from regulatory factors (such as hypoxia, immune cell activity or cytokine expression) are highlighted. In particular, malignant astrocytoma such as glioblastoma multiforme (GBM) is characterized by high heterogeneity at both intra- and intertumoral levels leading to more aggressive tendencies (2–6).

Previous studies have noted that the tumor microenvironment (TME) contains a host of tumor-associated macrophages (TAMs), which contribute to intertumoral diversity and are closely correlated with malignant phenotype of glioblastoma (6–8). TAMs functionally interact with both neoplastic and non-neoplastic cells within the milieu to have a profound impact on malignant progression (9, 10). According to the immunomodulatory properties, TAM is divided into pro-immune M1 type and pro-tissular M2 type (11), where M1 phenotype is responsible for inflammation and immune regulation; conversely, M2 phenotype is associated with tissue repair, cytoskeletal remodeling, angiogenesis, and immunosuppression (12, 13). Some scientists have reported mixed populations of both M1 and M2 macrophages in TAM distribution patterns; in fact, TAMs display highly plastic properties of M1/M2 switching in response to microenvironment cues (14–16). The neoplastic microenvironment strongly polarizes microglia/macrophages toward the M2 phenotypes, weakening the immune system to recognize and fight tumor cells (17–20). M2 macrophages are subdivided into M2a, M2b M2c and M2d subtypes, which contribute to tumor heterogeneity and plasticity (21). M2a is an alternatively activated macrophage, activated by interleukin (IL)-4, IL-13 or fungal and helminth infections; M2b belongs to type 2 macrophage or known as immune-regulatory macrophage, polarized by IL1 receptor ligands or LPS plus immune complexes; M2c is defined as deactivated macrophage responsive to IL-10, transforming growth factor-beta (TGF-beta) and glucocorticoids; M2d acts as switching macrophage or angiogenic “M2-like” phenotype upon stimulation by IL-6 and adenosine (21–24). The populations of CD163+ M2 macrophages have been shown to be enriched in high-grade gliomas, and their performance is inversely related to patient survival (25–29).

Current evidence has proven that glioma cells secrete CXC motif chemokine ligand 13 (CXCL13), which contributes to tumor immunity within the microenvironment (30–34). CXCL13, a 10 kDa CXC chemokine also known as B-cell chemoattractant-1 (BCA-1), physiologically mediates B cell mobilization and lymphoid tissue architecture (35, 36). CXCL13 specifically binds to CXC chemokine receptor type 5 (CXCR5) expressed by a specific subset of T cells, causes homing of lymphocytes to the lymphoid follicles while promoting antibody production (37, 38). It is absent in the normal central nervous system (CNS) and localizes to infiltrating immune cells in CNS inflammation (39). Under neoplastic conditions, CXCL13/CXCR5 axis acts on prolonged activation of oncogenic kinases and signaling that significantly contributes to organize cellular cluster formation (40–42). On the other hand, cancer cells secrete CXCL13 to immune cells, which are capable of production of cytokines directly promoting tumor progression and linking immune suppression (31, 43, 44). CXCL13- expressing malignant B cells have shown increased resistance against TNFα-mediated apoptosis (45, 46). Of note, CXCL13 through IL-10 induction promotes tumor macrophages in tissues that tend to develop in M2c type (47, 48).

However, the expression pattern and clinical significance of CXCL13 in human astrocytoma are still unclear, while the association between CXCL13 and M2 activation in astrocytoma also remains to be clarified. The goal of this study is to substantiate the clinicopathological significances for CXCL13 and M2 patterns (CD163+) in astrocytoma and to compare the potential utility of CXCL13 and CD163 as diagnostic biomarkers, alone and in combination, which discriminates between the different grades of astrocytoma (grades II, III, IV). With further analysis of CXCL13 expression and CXCL13/CD163 co-expression, the clinical parameters and prognostic factors were discussed, and their immunomodulatory influences in human astrocytoma were evaluated.

## Materials and Methods

### TCGA Dataset Analysis

*CXCL13* expression in the prognostic assessment of human gliomas was explained from the bioinformatics analysis of the TCGA low-grade glioma and glioblastoma (GBMLGG) dataset, which is composed of TCGA brain low-grade glioma (LGG) and glioblastoma multiforme (GBM) datasets. Astrocytoma, oligodendroglioma, and oligoastrocytoma were included in the LGG group, while the GBM group consisted of patients with glioblastoma multiforme. The level 3 data of exon expression and DNA methylation were downloaded by the University of California Santa Cruz (UCSC) Xena browser (https://xenabrowser.net). Glioma cases without exome sequencing or methylation data were excluded. 695 available cases were included in the *CXCL13* exon analysis, and 681 samples were identified as qualified samples for *CXCL13* methylation analysis.

*CXCL13* exon profiling was measured using the Illumina HiSeq 2000 RNA sequencing platform, provided by the TCGA Genome Characterization Center at the University of North Carolina. Four exons were included in this dataset: chr4:78432907–78432942, chr4:78526978–78527083, chr4:78528857–78528989, chr4:78531768–78531848. The transcription estimate at the exon level is shown by the RPKM value (reads per kilobase of exon model per million mapped reads). The average RPKM value from the four exons is reported as *CXCL13* expression. *CXCL13* DNA methylation profiling was performed using the Illumina Infinium Human Methylation 450 platform by the Johns Hopkins University and University of Southern California TCGA genome characterization center. Four methylation probes were included in this dataset: cg17001652, cg12020230, cg01134794, and cg06662476. DNA methylation beta value of each array probe is a continuous variable between 0 and 1, recorded through Bead Studio software, that is, the intensity ratio between the methylated beads and the binding site. *CXCL13* methylation status was evaluated by the average beta value of four methylation probes.

### Tissue Samples

The analysis was conducted retrospectively. To investigate whether prognosis would be related to the patient outcome, 112 astrocytoma patients were enrolled from the Cancer Center of Kaohsiung Medical University Hospital (KMUH); then, the astrocytoma was classified into grades II, III and IV with clinicopathological parameters, age, gender, WHO grade, tumor size, recurrence, survival rate and IDH1 mutation being selected. The authors reviewed the surgical pathology reports of patients diagnosed with astrocytoma, which included available data for tumor tissue and clinical follow-up while clinical and pathological data were obtained from the cancer registry and medical records. The study protocol was approved by the Institutional Review Board of KMUH (KMUHIRB-E(I)-20190188).

### Immunohistochemistry (IHC)

3 μm paraffin-embedded sections were de-paraffinized in xylene and dehydrated through the grading alcohol. Then, antigen retrieval was performed in 0.1 M citrate buffer (pH 6.0) at 121°C for 10 min. 3% hydrogen peroxide (H_2_O_2_) was used to block endogenous peroxidase activity and incubated for 5 min at room temperature. Sections were incubated with primary antibodies, CD163 (NCL-L-CD163, Leica Biosystems, United Kingdom), CXCL13 (MAB801, R&D Systems, Abingdon, United Kingdom), for 1 h at room temperature. Antigen-antibody complexes were visualized by DAKO REAL Envision detection system, peroxidase/DAB, rabbit/mouse (Dako, Glostrup, Denmark). Finally, sections were counterstained with hematoxylin and mounted. DoubleStain IHC Kit: Mouse and Rabbit on human tissue (DAB and AP/Red) (ab210059, Abcam, Cambridge, MA, United States) was used to assess the levels of two different antigens from the same tissue in immunohistochemical staining.

### Evaluation of IHC

The proportion of stained cells and the staining intensity of CXCL13 and CD163 were utilized to evaluate immune activity. The expression of CXCL13 and CD163 were evaluated by the degree of immunopositive cytoplasm and plasma membrane. CXCL13 staining was evaluated by the ratio score of positively stained cells (0, none; 1, <10%; 2, 10–50%; 3, >50%), and the intensity score of staining intensity (0, no staining; 1, weak; 2, medium; 3, strong). The product of the proportion of stained cells and staining intensity is the staining index. The total score ranges from 0 to 9, where 0–4 is defined as low expression and 6–9 is defined as high expression (Supplementary Table S1). CD163 expression was assessed by the average of stained cell frequency (0, <10 cells; 1, 10–49 cells; 2, 50–100 cells; 3, >100 cells) and distribution (perivascular or scattered in the parenchyma) in five randomly selected high power fields (×400 magnification) (49). The semi-quantitative scoring system includes four categories: 0, 1, 2 and 3, comparisons were made by two categories of CD163 staining scores: low expression (including the above 0 and 1) and high expression (including the above 2 and 3) (Supplementary Table S1).

### Statistical Analysis

Descriptive statistics estimated for the study population included means with corresponding standard deviations (SD), medians with corresponding ranges, and proportions, together with 95% confidence intervals (95% CI). Associations between target proteins (CXCL13 and CD163) and clinicopathological parameters were analyzed by chi-square test; the strength of the relationship between two variables were measured using Pearson’s correlation; survival and hazard functions were illustrated by Kaplan–Meier survival curve; and survival was compared between groups by two-sided log-rank test. Cox proportional hazard model was used to examine risk factors related to survival after adjusting for other factors, with risk factors including gender, age, grade, and recurrence. Statistical analyses were performed with SAS 9.3 (SAS Institute, Cary, NC, United States).

## Results

### CXCL13 Expression Serves as a Prognostic Biomarker for Glioma Outcomes

**FIGURE 1:**
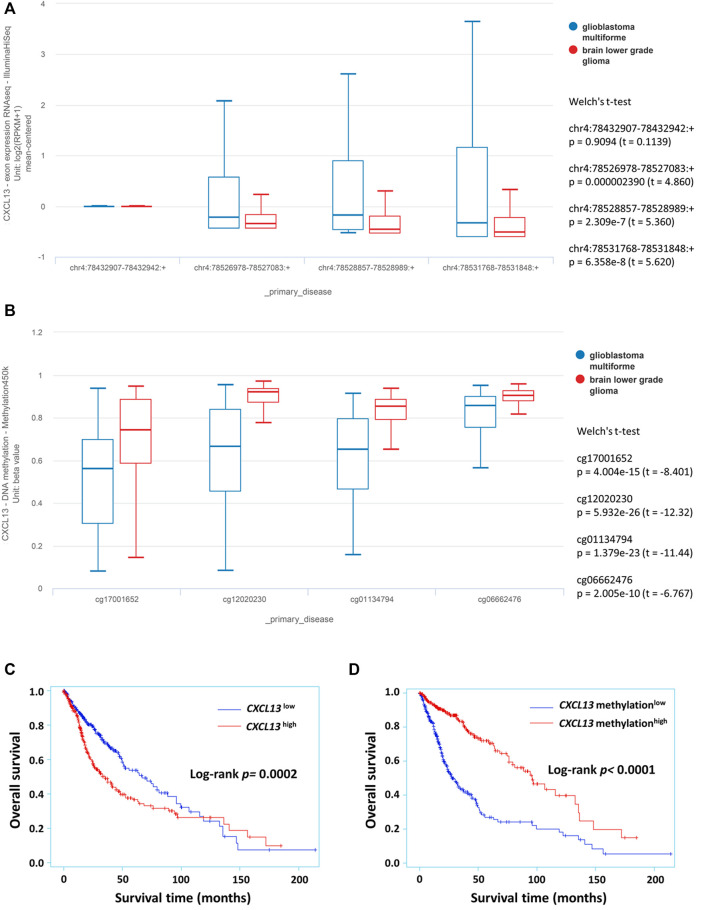
The prognostic potential of *CXCL13* in glioma identified from TCGA GBMLGG dataset. The expression of *CXCL13* exon **(A)** and DNA methylation **(B)** in glioma subgroup were analyzed by the UCSC Xena platform. Kaplan–Meier curves were performed with exon expression **(C)** and DNA methylation **(D)** that showed the relationship between *CXCL13* gene expression with overall survival in glioma. *p* < 0.05 was considered statistically significant. Abbreviations: TCGA: The Cancer Genome Atlas; UCSC: University of California Santa Cruz.

Public database analysis from the TCGA GBMLGG cohort was used to evaluate the *CXCL13* expression in varying degree of malignant glioma. The prognostic potential of *CXCL13* gene in gliomas was verified by 529 LGGs and 166 GBMs in exon expression profiles and DNA methylation patterns. As described in the method section, the four exons and four methylation probes of *CXCL13* are used for gene expression analysis and conducted by UCSC Xena (Figures 1A,B). Bioinformatics analysis revealed that *CXCL13* exhibited greater expression in the GBM group (chr4:78526978–78527083:+, chr4:78528857–78528989:+, chr4:78531768–78531848:+, all *p* < 0.0001, Welch’s *t*-test; Figure 1A). On the contrary, lower *CXCL13* methylation was observed in GBMs (all *p* < 0.0001, Welch’s *t*-test; Figure 1B).

Further, the associations of *CXCL13* expression and methylation status with overall survival were analyzed by Kaplan–Meier curves. *CXCL13* expression is divided into low (−) and high (+) groups, depending on the median of exon expression profiles (0.0755) and methylation profiles (0.8231). *CXCL13* as an available prognostic biomarker for glioma outcome was illustrated by Kaplan-Meier curves (exon, *p* = 0.0002; methylation, *p* < 0.0001; Figures 1C,D). High level of *CXCL13* was strongly correlated with age (*p* < 0.0001), WHO grade (*p* < 0.0001), patient survival (*p* = 0.0012, Supplementary Table S2). *CXCL13* methylation was associated with age (*p* < 0.0001), grade (*p* < 0.0001), patient survival (*p* < 0.0001), and IDH1 mutation (*p* = 0.0309; Supplementary Table S2).

### Patient Characteristics

A total of 112 cases of astrocytoma were enrolled in this study, including 45 females and 67 males (Table 1). The average age is 50.61 ± 17.73 years, and the median age is 52.50 years (range, 20–83 years). A subgroup of tumor size is defined relative to 2 cm and the mean tumor size ±SD, 2.85 ± 2.00 cm. The mean (±SD) follow-up time of this cohort was 22.66 (±19.43) months. Forty patients died during the follow-up period. Notably, 45 (40.18%) patients had the highest-grade carcinomas (WHO grade IV), 51 (45.54%) patients had IDH1 mutations, and 57 (50.89%) patients had recurrence.

**TABLE 1:** Correlation between CXCL13, CD163, CXCL13/CD163 and clinicopathological parameters in astrocytoma.

Parameters	*n*	CXCL13		*p* value	CD163		*p value*	CXCL13/CD163		*p* value
		Low(%)	High(%)		Low(%)	High(%)		Low(%)	High(%)	
Gender				0.1941			0.4086			0.3396
Female	45	31(44.93)	14(32.56)		21(44.68)	24(36.92)		34(43.04)	11(33.33)	
Male	67	38(55.07)	29(67.44)		26(55.32)	41(63.08)		45(56.96)	22(66.67)	
Age				0.1631			0.1194			0.8880
≤45 years	43	23(33.33)	20(46.51)		22(46.81)	21(32.31)		30(37.97)	13(39.39)	
>45 years	69	46(66.67)	23(53.49)		25(53.19)	44(67.69)		49(62.03)	20(60.61)	
WHO grade				0.0002			<0.0001			<0.0001
II	32	27(39.13)	5(11.63)		22(46.81)	10(15.38)		30(37.97)	2(6.06)	
III	35	24(34.78)	11(25.58)		18(38.30)	17(26.15)		28(35.44)	7(21.21)	
IV	45	18(26.09)	27(62.79)		7(14.89)	38(58.46)		21(26.58)	24(72.73)	
Tumor size				0.5969			0.0939			0.3027
<2 cm	46	27(39.13)	19(44.19)		15(31.91)	31(47.69)		30(37.97)	16(48.48)	
≧2 cm	66	42(60.87)	24(55.81)		32(68.09)	34(52.31)		49(62.03)	17(51.52)	
Recurrence				0.6645			0.9754			0.7418
Absent	55	35(50.72)	20(46.51)		23(48.94)	32(49.23)		38(48.10)	17(51.52)	
Present	57	34(49.28)	23(53.49)		24(51.06)	33(50.77)		41(51.90)	16(48.48)	
Survival status				0.0017			0.0527			0.0007
survived	32	27(39.13)	5(11.63)		18(38.30)	14(21.54)		30(37.97)	2(6.06)	
died	80	42(60.87)	38(88.37)		29(61.70)	51(78.46)		49(62.03)	31(93.94)	
IDH1 mutant				0.0008			0.0314			0.0002
Negative	61	29(42.03)	32(74.42)		20(42.55)	41(63.08)		34(43.04)	27(81.82)	
Positive	51	40(57.97)	11(25.58)		27(57.45)	24(36.92)		45(56.96)	6(18.18)	

### CXCL13 and CD163 Immunophenotype in Astrocytoma Tissues and Their Associations With Clinicopathologic Features

**FIGURE 2:**
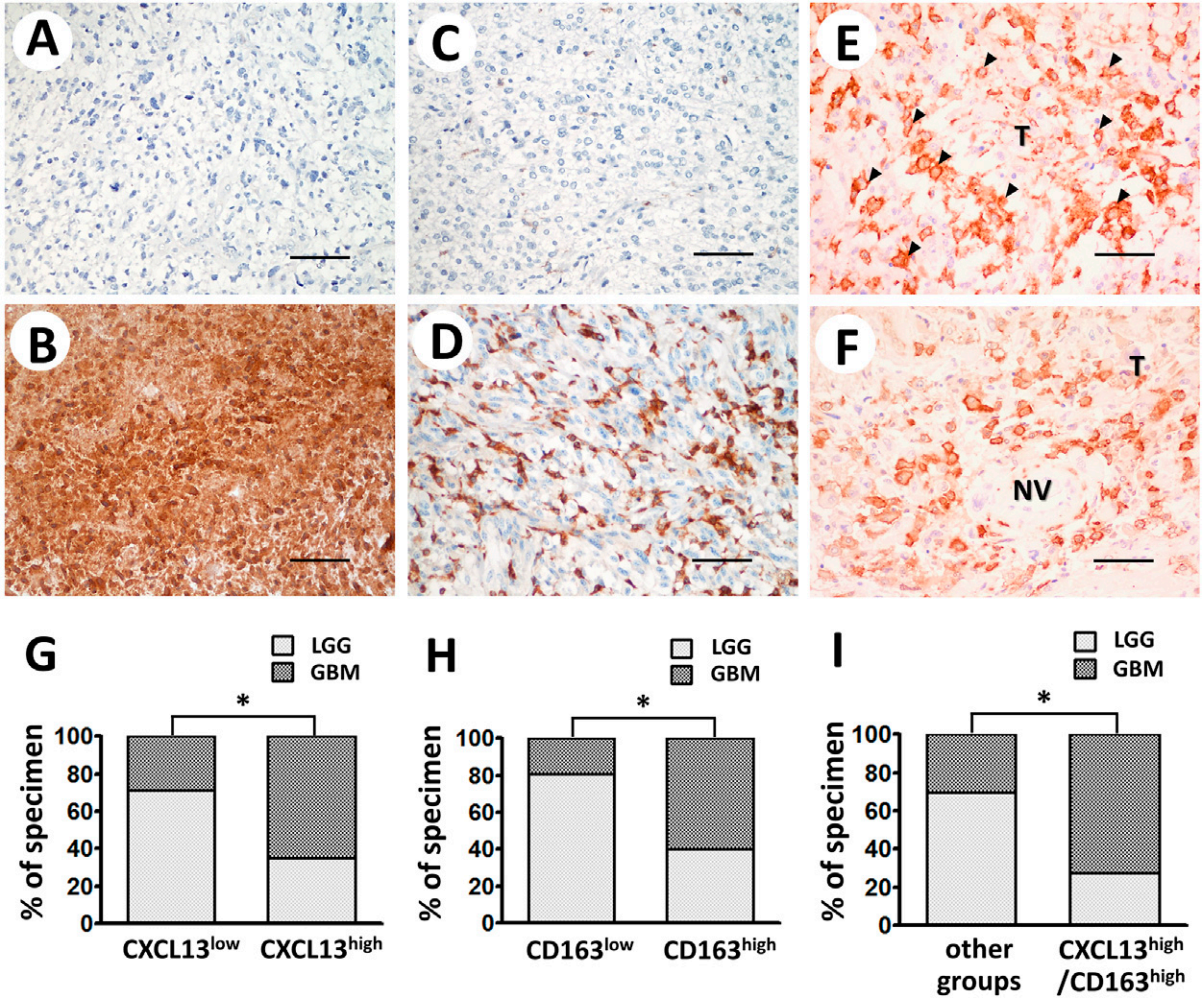
Representative immunostaining of CXCL13 and CD163 in human astrocytoma. Immunoreactivity of CXCL13 **(A,B)** and CD163 **(C,D)** was classified as low or high expression according to staining observed in cytoplasm and plasma membrane. **(E,F)** Double-staining of CXCL13 protein in tumor tissues (red cytoplasmic staining) and CD163+ M2 (brown plasma membrane staining) demonstrated co-occurrence of CXCL13 and CD163 in astrocytoma tissues. Black arrows indicate the CD163+ M2 macrophages infiltrate in malignant tissues (×200 magnification, scale bar 100 μm). Immunoreactivity percentages of CXCL13 **(G)**, CD163 **(H)**, and CXCL13/CD163 **(I)** phenotypes were observed in lesions. LGG subgroup is depicted as light gray columns, whereas GBM subgroup is shown as dark gray columns. Chi-square test was used for statistical analysis. *p < 0.05 was considered statistically significant. Abbreviations: LGG, lower grade glioma; GBM, glioblastoma multiforme; T, tumor tissue; NV, neovasculature.

CXCL13 and CD163 expressions in astrocytoma tissues were displayed by immunohistochemical staining. Immunoreactivity of CXCL13 and CD163 was determined based on cytoplasmic and plasma membrane staining (low or high expression of CXCL13, Figures 2A or B, respectively; low or high expression of CD163, Figures 2C or D, respectively). Significantly high levels of CXCL13 and CD163 have been observed in some malignant tissues (Figures 2B,D). In CXCL13/CD163 double staining, CXCL13 was stained red in the cytoplasm of both tumor cells and TAMs, whereas CD163 was visible in brown plasma membrane staining of M2 macrophages. Intense M2 infiltration was observed in high-grade astrocytoma tissue and surrounding neovasculature by double staining (Figures 2E,F). The significant increases in CXCL13, CD163, and CXCL13/CD163 immunoreactivity of astrocytoma tissues were found in the apparently aggressive GBM subgroups (CXCL13, *p* = 0.0002; CD163, *p* < 0.0001; CXCL13/CD163, *p* < 0.0001; Figures 2G–I). Table 1 shows the associations of CXCL13, CD163, and CXCL13/CD163 phenotypes in 112 astrocytoma patients with various clinicopathological parameters. Increased level of CXCL13 was detected in 43 (38.39%) patients, and it was strongly correlated with grade (*p* = 0.0002), patient survival (*p* = 0.0017), and IDH1 mutation (*p* = 0.0008; Table 1). High expression of CD163 was associated with tumor grade (*p* < 0.0001) and IDH1 mutation (*p* = 0.0314; Table 1). There were positive associations between CXCL13/CD163 and grade (*p* < 0.0001), patient survival (*p* = 0.0007), and IDH1 mutation (*p* = 0.0002; Table 1). When CD163 and CXCL13 coexisted, it significantly affected patient survival, which was consistent with the hypothesis that CXCL13 showed enhanced M2 regulation of tumor immune escape. Besides, the results of correlation coefficients indicated CXCL13 had strong correlation with CD163+ M2 distribution (*p* = 0.0013) and IDH1 mutation (*p* = 0.0007; Table 2). Pearson correlation also confirmed that CD163 expression also correlated with IDH1 mutation (*p* = 0.0315; Table 2).

**TABLE 2:** Pearson analysis of the relation between CXCL13, CD163 and IDH1 mutation.

Parameters	CXCL13	*p* value	CD163	*p* value	IDH1 mutation	*p* value
CXCL13	1.00000	-	0.29927	0.0013	−0.31631	0.0007
CD163	0.29927	0.0013	1.00000	-	−0.20338	0.0315
IDH1 mutation	−0.31631	0.0007	−0.20338	0.0315	1.00000	-

### The Prognostic Value of CXCL13, CD163 and CXCL13/CD163 Coexpression in Astrocytoma

**FIGURE 3:**
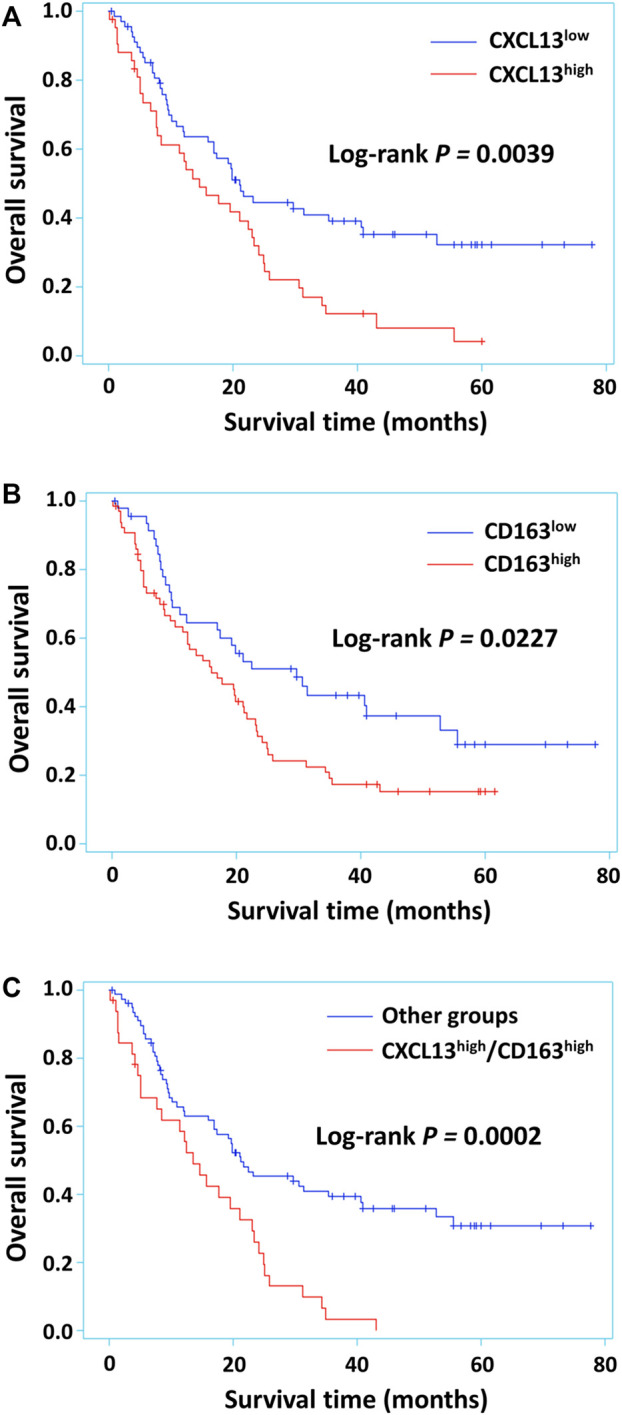
Kaplan-Meier survival curves for astrocytoma patients with different levels of CXCL13, CD163 or CXCL13/CD163 co-expression. CXCL13 **(A)** and CD163 **(B)** alone expression had significant effects on survival in 112 astrocytoma patients. Survival in patients that had tumors with CXCL13/CD163 co-expression **(C)** in comparison with other phenotypes was observed. *p* < 0.05 was considered statistically significant.

The Kaplan-Meier survival curve was used to evaluate the relationship between CXCL13, CD163 and patient survival of astrocytoma. Poor prognosis of the patients were found in the high performance of CXCL13, CD163 and CXCL13/CD163 (*p* = 0.0039, *p* = 0.0227, and *p* = 0.0002; Figure 3). Univariate and multivariate logistic analyses were used to observe the independent prognostic clinicopathological indicators of survival in astrocytoma (Table 3). The results of univariate logistic analysis showed that high expression of CXCL13 was significantly associated with poor overall survival (HR = 1.897, 95% CI: 1.218–2.954, *p* = 0.0046; Table 3). Multivariate Cox regression analysis including age, tumor size, gender, and recurrence indicated that high level of CXCL13 may not be a significant predictor of OS (HR = 1.569; 95% CI, 0.974–2.527; *p* = 0.0642; Table 3). Furthermore, CXCL13/CD163 phenotype revealed significant association between co-expression of these two proteins and patient outcomes in univariate analysis (HR = 2.369, 95% CI: 1.491–3.764, *p* = 0.0003; Table 3). After adjusting for parameters such as gender, age, grade, and recurrence, CXCL13/CD163 coexpression was also regarded as an independent prognostic indicator of patient survival in astrocytoma (HR = 1.682, 95% CI: 1.032–2.740, *p* = 0.0368; Table 3).

**TABLE 3:** Univariate and multivariate analysis of overall survival in 112 patients with astrocytoma.

Parameters	Univariate		Multivariate					
	HR (95% CI)	*p*-value	CXCL13	*p*-value	CD163	*p*-value	CXCL13/CD163	*p*-value
			HR (95% CI)		HR (95% CI)		HR (95% CI)	
CXCL13	1.897 (1.218–2.954)	0.0046	1.569 (0.974–2.527)	0.0642	-	-	-	-
CD163	1.693 (1.071–2.678)	0.0243	-	-	1.216 (0.756–1.956)	0.4200	-	-
CXCL13/CD163	2.369 (1.491–3.764)	0.0003	-	-	-	-	1.682 (1.032–2.740)	0.0368
Gender (male = 1)	1.507 (0.950–2.389)	0.0814	1.473 (0.920–2.357)	0.1065	1.575 (0.991–2.504)	0.0549	1.502 (0.942–2.395)	0.0877
Age	1.637 (1.027–2.609)	0.0382	1.381 (0.827–2.305)	0.2174	1.198 (0.729–1.968)	0.4757	1.286 (0.785–2.105)	0.3178
Grade	3.670(1.972–6.827)	<0.0001	3.336 (1.715–6.488)	0.0004	3.656 (1.882–7.103)	0.0001	3.212 (1.633–6.315)	0.0007
Recurrence	0.922 (0.592–1.436)	0.7188	0.791 (0.490–1.276)	0.3362	0.762 (0.473–1.227)	0.2634	0.812 (0.505–1.304)	0.3882

*HR* hazard ratio (HR) and 95% CI were calculated using Cox regression analysis.

## Discussion

This study is the first to point out that CXCL13 either alone or when co-expressed with M2 pattern CD163 could be a predictive marker of astrocytoma progression and patient outcome. CXCL13 acts as a B-cell chemoattractant in lymphoid neogenesis and is widely involved in the autoimmune pathogenesis and lymphoproliferative disorders. Currently, CXCL13 has been found overexpressed in malignant tissues and coordinates tumor progression by modulating cell-cell interactions and lymphocyte recruitment in the TME (33, 42, 50). TAMs are the most abundant non-neoplastic cell population in refractory glioma that lead to tolerogenic TME and therapeutic resistance (51, 52). TAM infiltration in the TME is stimulated by tumor-derived cytokines and chemoattractants, whereby TAM creates a milieu conducive to glioma progression through the secretion of pro-tumorigenic factors and anti-inflammatory cytokines (53, 54). The effect of anti-inflammatory phenotype polarization such as M2 can originate from cell infiltration, angiogenesis, and immune evasion. To enable M2 for exerting its tumor immunomodulatory ability, identifying the biomarkers CD163 has indeed been performed in recent experiments (55, 56). As our previous findings, CD163+ M2 infiltration in glioma tissues was progressively correlated with tumor malignancy and worse outcome (57). In particular, CXCL13 through IL-10 induction promotes TAMs that tend to M2c activation, leading to poor immunogenicity and immunosuppression (48).

Previous studies have indicated that the prognostic role of CXCL13 seems to be tumor-type dependent. In this study, the prognostic potential of *CXCL13* gene expression was demonstrated by 695 glioma samples from TCGA GBMLGG datasets. *CXCL13* expression was positively related to poor survival rate, but the DNA methylation status was inversely correlated with patient outcomes. Decreased levels of DNA methylation occur in glioma patients with poor outcomes, which imply that demethylation acts as epigenetic modification to enhance *CXCL13* expression. Consistent with the *in silico* analysis, the present immunohistochemistry result indicated a strong relationship between CXCL13 and poor prognosis of the glioma (*p* = 0.0039, Figure 3A), supporting our initial hypothesis. In this study, 43 out of 112 astrocytoma patients showed high CXCL13 phenotype, which closely paralleled earlier reports that malignant neoplasms expressed high level of CXCL13 (32). The regression results also confirmed CXCL13 as an independent prognostic indicator for human astrocytoma. In addition, both CXCL13 and CD163 were inversely associated with IDH1 mutations (CXCL13, *p* = 0.0007; CD163, *p* = 0.0315, Table 2), implying that fewer M2 TAMs were present in IDH-mutant astrocytomas compared to wild-type. These results are consistent with previous reports that less TAM infiltration leads to pro-inflammatory effects and also tends to favor mesenchymal features contributing to better prognosis in IDH-mutated gliomas (58, 59).

Although CXCL13 has been considered as a predictive marker for astrocytoma, the influence of CXCL13-related M2 immunity during astrocytoma progression has not been evaluated. This study further elaborated the clinicopathological significance of CXCL13 co-expressed with CD163, providing a link between CXC chemokine and M2 immunity in human astrocytoma. We observed CXCL13/CD163 coexpression was correlated with grade, survival, and IDH1 mutations. There is the significantly worse overall survival outcome in CXCL13/CD163 coexpression (*p* = 0.0002, Figure 3C; *p* = 0.0003, *Univariate analysis*, Table 3)*.* CXCL13/CD163 coexpression, but not both proteins alone, is associated with overall survival independently from clinicopathological factors (CXCL13, *p* = 0.0642; CD163, *p* = 0.4200; CXCL13/CD163, *p* = 0.0368, Multivariate analysis, Table 3). These results suggested that CXCL13-mediated immunomodulation of M2 has a considerable impact on the prognosis of astrocytoma.

In conclusion, CXCL13 expression is associated with poor outcome in astrocytoma; crucially, CXCL13 might promote M2 infiltration into malignant lesions and the surrounding neovasculature. As with recent studies, CXCL13/CD163 double staining results confirmed that CXCL13 is secreted by a variety of cells within the TME, including tumor cells and tumor-infiltrating immune cells. It specifically binds to the corresponding receptor CXCR5, directly regulating tumor progression or indirectly modulating adaptive immune responses (50, 60, 61). Notably, CXCL13 drives M2c activation through IL-10 induction and results in poor immunogenicity and immunosuppression (48, 62, 63). M2c enhances glioma cell-induced immune tolerance through cytokine secretion and crosstalk with infiltrating effector cells, thereby forming an immunosuppressive microenvironment (7, 53, 64). This means that CXCL13 inhibition can destroy tumor cells, and its combination therapy may improve patient outcomes. These results provide an innovative characterization of CXCL13 distribution and M2 infiltration across the astrocytoma subgroups, setting the basis for diagnostics and therapeutic insights that may contribute to the application of immunotherapies in human astrocytoma.

## Data Availability

The original contributions presented in the study are included in the article/Supplementary Material, further inquiries can be directed to the corresponding author.
