# Supplementary material for: The Diagnostic Significance of CXCL13 in M2 Tumor Immune Microenvironment of Human Astrocytoma
Source: Pathol Oncol Res. 2022 Apr 28;28:1610230. doi: 10.3389/pore.2022.1610230 (PMC9095826; doi:10.3389/pore.2022.1610230)
Supplement: Supplementary file 1 [file Table1.docx]

Table S1 Score systems for evaluation of CXCL13 and CD163 immunostaining

| **CXCL13 scoring** | | | |
| --- | --- | --- | --- |
| Proportion of stained cells | Staining intensity | Score | Staining index |
| none | no staining | 0 | 0-4: Low expression  6-9: High expression |
| <10% | weak | 1 |  |
| 10-50% | medium | 2 |  |
| >50% | strong | 3 |  |

* Staining index = Proportion of stained cells * Staining intensity

| **CD163 scoring** | | | |
| --- | --- | --- | --- |
| Frequency of stained cells | Distribution | Score | Staining index |
| <10 cells | Only perivascular | 0 | 0, 1: Low expression  2, 3: High expression |
| 10-49 cells | Perivascular and scattered  in the parenchyma | 1 |  |
| 50-100 cells |  | 2 |  |
| >100 cells |  | 3 |  |

* The positive cell frequency and distribution were observed in five randomly selected high power fields (x400).
