# Supplementary material for: The Diagnostic Significance of CXCL13 in M2 Tumor Immune Microenvironment of Human Astrocytoma
Source: Pathol Oncol Res. 2022 Apr 28;28:1610230. doi: 10.3389/pore.2022.1610230 (PMC9095826; doi:10.3389/pore.2022.1610230)
Supplement: Supplementary file 2 [file Table2.docx]

Table S2 Correlation between CXCL13 expression and clinicopathological parameters in gliomas from the TCGA datasets

| **Parameters** |  | ***CXCL13*** | | *p* value |  | ***CXCL13 methylation*** | | *p value* |
| --- | --- | --- | --- | --- | --- | --- | --- | --- |
|  | n | Low(%) | High(%) |  | n | Low(%) | High(%) |  |
| **Gender**  Female  Male | 297  398 | 148(42.65)  199(57.35) | 149(42.82)  199(57.18) | 0.9650 | 301  380 | 147(43.36)  192(56.64) | 154(45.03)  188(54.97) | 0.6615 |
| **Age**  ≤45 years  >45 years | 346  349 | 201(57.93)  146(42.07) | 145(41.67)  203(58.33) | <0.0001 | 342  339 | 122(35.99)  217(64.01) | 220(64.33)  122(35.67) | <0.0001 |
| **WHO grade**  II  III  IV | 259  270  166 | 143(41.21)  153(44.09)  51(14.70) | 116(33.33)  117(33.62)  115(33.05) | <0.0001 | 259  270  152 | 69(20.35)  138(40.71)  132(38.94) | 190(55.56)  132(38.60)  20(5.85) | <0.0001 |
| **Recurrence**  Absent  Present | 518  177 | 253(72.91)  94(27.09) | 265(76.15)  83(23.85) | 0.3271 | 501  179 | 248(73.16)  91(26.84) | 253(74.19)  88(25.81) | 0.7588 |
| **Survival status**  survived  died | 429  266 | 235(67.72)  112(32.28) | 194(55.75)  154(44.25) | 0.0012 | 442  239 | 174(51.33)  165(48.67) | 268(78.36)  74(21.64) | <0.0001 |
| **IDH1 mutant**  Negative  Positive | 342  95 | 196(78.09)  55(21.91) | 146(78.49)  40(21.51) | 0.9188 | 308  161 | 137(71.35)  55(28.65) | 171(61.73)  106(38.27) | 0.0309 |
